# Prevalence of mental disorders among elderly men: a systematic review and meta-analysis

**DOI:** 10.1590/1516-3180.2019.0454.R1.16012020

**Published:** 2020-06-01

**Authors:** Genef Caroline Andrade Ribeiro, Walbert de Andrade Vieira, Álex Moreira Herval, Renata Prata Cunha Bernardes Rodrigues, Bernardo Antonio Agostini, Carlos Flores-Mir, Carlos Eduardo Palanch Repeke, Luiz Renato Paranhos

**Affiliations:** I MSc. Speech Therapist, Speech Therapy Department, Universidade Federal de Sergipe (UFS), Lagarto (SE), Brazil.; II DDS. Dentist and Master’s Student, Department of Restorative Dentistry, Endodontics Division, Faculdade de Odontologia de Piracicaba (FOP), Universidade Estadual de Campinas (UNICAMP), Piracicaba (SP), Brazil.; III PhD. Dentist, Department of Preventive and Community Dentistry, School of Dentistry, Universidade Federal de Uberlândia (UFU), Uberlândia (MG), Brazil.; IV MSc. Dentist, Department of Preventive and Community Dentistry, School of Dentistry, Universidade Federal de Uberlândia (UFU), Uberlândia (MG), Brazil.; V PhD. Dentist, Postgraduate Program on Dentistry, Faculdade Meridional (IMED), Passo Fundo (RS), Brazil.; VI DSc. Dentist, Division of Orthodontics, School of Dentistry, University of Alberta, Edmonton (AB), Canada.; VII PhD. Dentist, Postgraduate Program of Health Science, Universidade Federal Sergipe (UFS), Lagarto (SE), Brazil.; VIII PhD. Dentist, Department of Preventive and Community Dentistry, School of Dentistry, Universidade Federal de Uberlândia (UFU), Uberlândia (MG), Brazil.

**Keywords:** Aged, Men, Mental disorders, Depression, Schizophrenia, Mental disease, Elderly men, Older men

## Abstract

**BACKGROUND:**

Elderly men have been characterized as a group vulnerable to suicide, motivated by loneliness, loss of loved ones and feelings of uselessness to family members.

**OBJECTIVES:**

To ascertain the prevalence of different mental disorders among elderly men who attempted suicide.

**DESIGN AND SETTING:**

Systematic review of observational studies developed as a result of a partnership between two postgraduate schools (Lagarto and Uberlândia).

**METHODS:**

An electronic search was performed in eight electronic databases, including “grey literature”, in January 2019. Observational studies that assessed mental disorders among men older than 60 years who attempted suicide were eligible for inclusion.

**RESULTS:**

Among the disorders evaluated, mood disorders had the highest prevalence (42.0%; 95% confidence interval, CI: 31.0-74.0%; I^2^: 0.0%; P = 0.763), followed by substance use-related disorders (41.0%; 95% CI: 8.0-74.0%; I^2^: 96.4; P < 0.001) and, lastly, schizophrenic disorders (5.0%; 95% CI: 0.0%-14.0%; I^2^: 80.3%; P = 0.024).

**CONCLUSIONS:**

It seems that mood disorders and substance use-related disorders are quite prevalent among elderly men with mental disorders who attempted suicide. It is important to consider the role of healthcare services in making early diagnoses of mental disorders among elderly men, in order to diminish the chances of suicide attempts among them.

SYSTEMATIC REVIEW REGISTRATION: CRD42018105981.

## INTRODUCTION

The proportion of the world population corresponding to elderly people has grown extensively and it currently represents 12.3% of the total population. It has been estimated that the prevalence of this age group may reach 21.5% by 2050.^1^ Countries such as France, England and Canada are already classified as elderly countries, considering that more than 14% of their populations are older than 60 years.^2^ This trend is also starting to be noticed even in emerging countries like Brazil, where elderly people account for 12.5% of the population.^3^ Advances in medicine, lifestyle changes, better educational conditions and better quality of life have been correlated with aging populations.^4^

Along with the growth in the elderly population, the suicide rate among the elderly has also increased over the last few years.^5^^,^^6^ In 2015, suicide was the second commonest cause of death among the elderly, only behind chronic diseases.^1^ In overall terms, suicide kills more than homicides and wars together.^7^ European countries and Japan are the leaders in this ranking.^8^

Suicide is such a complex and multifactorial phenomenon that its occurrence cannot be attributed to any single characteristic or event.^9^ Among the groups that are more vulnerable to suicide, the proportion in the elderly population is increasing the most.^10^^,^^11^ The main risk factors in this age group are systemic diseases, loss of family members, impossibility of maintaining the standard of living and mental disorders,^12^^,^^13^ along with genetic factors that are potentially involved.^14^^,^^15^

Among elderly men, the number of suicides can be four times higher than among women.^9^ The main cause of suicide among elderly men seems to be mental disorders.^16^^,^^17^ We did not identify any evidence-based synthesis of such data.

## OBJECTIVE

The aim of this study was to perform a systematic review of the literature on the prevalence of mental disorders among elderly men who attempted suicide. We sought to answer the following guiding question: “what types of mental disorders are more prevalent among elderly men who attempted suicide?”.

## METHODS

### Protocol and registration

This systematic review was conducted in accordance with the list of PRISMA-P statements (Preferred Reporting Items for Systematic Reviews and Meta-Analyses Protocols)^18^ and the MOOSE statements (Meta-Analyses of Observational Studies in Epidemiology),^19^ along with the Cochrane guidelines.^20^ The protocol for this systematic review was registered in the PROSPERO database (CRD42018105981).

### Study design and eligibility criteria

The systematic review was designed to answer the guiding question, through a population, variables and outcomes (PVO) strategy. In this, the population comprised elderly men with mental disorders, the variables analyzed were different types of mental disorders and the outcome was suicide attempts. Diagnoses for the health conditions presented by this population were considered in accordance with the codes of the International Classification of Diseases, 11^th^ revision (ICD-11).

The studies that were considered eligible were observational studies on men older than 60 years who attempted suicide (ICD-11: XE97V) and who were diagnosed as presenting any of the following: mental and behavioral disorders (ICD-11: 6); organic mental disorders including symptomatic ones (ICD-11: 6E6); schizophrenia, schizotypal and delusional disorders (ICD-11: 6A2); mood disorders (ICD-11: 6A8 and 6C4G.7); unspecified mental disorders (ICD-11: 6D1); or substance use-related disorders (ICD-11: 6C4Z). No restriction regarding year, language or publication status were imposed in this search.

The exclusion criteria were as follows: 1) studies not related to the stated objective; 2) studies that did not present segmented data for men and/or elderly people; 3) studies that dealt only with suicidal thoughts or suicide attempts; 4) review studies, brief communications, editorials, letters to the editor, case reports, theses, congress abstracts, books/book chapters, textbooks and technical reports; and 5) studies with a high risk of bias (low methodological quality).

### Sources of information and search strategies

The descriptors were selected from the Descriptors in Health Sciences (DeCS) and the Medical Subject Headings (MeSH). The databases used were LILACS, PubMed (including MEDLINE), SciELO, Scopus and Web of Science. OpenThesis, OATD and OpenGrey were used to partially capture the “grey literature”.

The descriptors selected were: “Elderly”, “Aged”, “Older”, “Elder”, “Man”, “Men”, “Male”, “Males”, “Suicide”, “Suicides”, “Suicidal”, “Mental disorders”, “Psychiatric Illness”, “Psychiatric diagnosis”, “Behavior disorders”, “Mood disorders”, “Affective disorders” and “Personality disorders”. The Boolean operators “AND” and “OR” were used to enhance the search strategy through several combinations (Table 1). The bibliographic search was performed in January 2019. The results obtained were exported to the Mendeley™ software (Elsevier™, Amsterdam, Netherlands), in which duplicates were removed electronically. The remaining results were exported to Microsoft Word™ 2010 (Microsoft™ Ltd, Washington, USA) and the remaining duplicates were removed manually.

**Table 1.** Search strategies in databases

Databases	Search strategy (January 2019)	Results
PubMed http://www.ncbi.nlm.nih.gov/pubmed	(“Elderly”[All Fields] OR “Aged”[All Fields] OR “Older”[All Fields] OR “Elder”[All Fields]) AND (“Man”[All Fields] OR “Men”[All Fields] OR “Male”[All Fields] OR “Males”[All Fields]) AND (“Suicide”[All Fields] OR “Suicides”[All Fields] OR “Suicidal”[All Fields]) AND (“Mental Disorders”[All Fields] OR “Psychiatric Illness”[All Fields] OR “Psychiatric Diagnosis”[All Fields] OR “Behavior Disorders”[All Fields] OR “Mood Disorders”[All Fields] OR “Affective Disorders”[All Fields] OR “Personality Disorders”[All Fields])	4,941
Scopus (Elsevier) http://www.scopus.com/	(“Elderly” OR “Aged” OR “Older” OR “Elder”) AND (“Men” OR “Males”) AND (“Suicide”) AND (“Mental Disorders” OR “Psychiatric Illness” OR “Psychiatric Diagnosis” OR “Behavior Disorders” OR “Mood Disorders” OR “Affective Disorders” OR “Personality Disorders”)	5,843
Web of Science (Clarivate Analytics) http://apps.webofknowledge.com/	((“Elderly” OR “Aged” OR “Older” OR “Elder”) AND (“Man” OR “Men” OR “Male” OR “Males”) AND (“Suicide” OR “Suicides” OR “Suicidal”) AND (“Mental Disorders” OR “Psychiatric Illness” OR “Psychiatric Diagnosis” OR “Behavior Disorders” OR “Mood Disorders” OR “Affective Disorders” OR “Personality Disorders”))	390
LILACS (Virtual Health Library) http://lilacs.bvsalud.org/	(“Elderly” OR “Aged”) AND (“Man” OR “Male”) AND (“Suicide”) AND (“Mental Disorders”) AND (instance:”regional”) AND ( db:(“LILACS”))	52
	tw:((“Older” OR “Elder”) AND (“Men” OR “Males”) AND (“Suicide”) AND (“Mental Disorders”)) AND (instance:”regional”) AND ( db:(“LILACS”))	3
	tw:((“Elderly” OR “Aged”) AND (“Man” OR “Male”) AND (“Suicide”) AND (“Psychiatric Illness “)) AND (instance:”regional”) AND ( db:(“LILACS”))	1
	tw:((“Elderly” OR “Aged”) AND (“Man” OR “Male”) AND (“Suicide”) AND (“Psychiatric Diagnosis”)) AND (instance:”regional”) AND ( db:(“LILACS”))	31
	tw:((“Elderly” OR “Aged”) AND (“Man” OR “Male”) AND (“Suicide”) AND (“Behavior Disorders”)) AND (instance:”regional”) AND ( db:(“LILACS”))	31
	tw:((“Elderly” OR “Aged”) AND (“Man” OR “Male”) AND (“Suicide”) AND (“Mood Disorders”)) AND (instance:”regional”) AND ( db:(“LILACS”))	9
SciELO http://www.scielo.org/	“Suicide” AND “Mental Disorders” AND “Elderly”	5
	“Suicide” AND “Mental Disorders” AND “Older”	7
	“Suicide” AND “Mental Disorders” AND “Aged”	11
	“Suicide” AND “Psychiatric Illness” AND “Aged”	0
	“Suicide” AND “Mood Disorders” AND “Aged”	7
	“Suicide” AND “Behavior Disorders” AND “Aged”	0
OpenThesis http://www.openthesis.org/	(“Aged” OR “Older” OR “Elder”) AND (“Male”) AND (“Suicide”) AND (“Mental Disorders” OR “Psychiatric Illness” OR “Psychiatric Diagnosis” OR “Behavior Disorders” OR “Mood Disorders” OR “Affective Disorders” OR “Personality Disorders”)	1,560
OATD https://oatd.org/	(“Aged” OR “Older” OR “Elder”) AND (“Male”) AND (“Suicide”) AND (“Mental Disorders” OR “Psychiatric Illness” OR “Psychiatric Diagnosis” OR “Behavior Disorders” OR “Mood Disorders” OR “Affective Disorders” OR “Personality Disorders”)	3
OpenGrey http://www.opengrey.eu/	(“Aged”) AND (“Male”) AND (“Suicide”) AND (“Mental Disorders”)	0
PsycNet (American Psychological Association) https://psycnet.apa.org/search/basic	((“Elderly” OR “Aged” OR “Older” OR “Elder”) AND (“Man” OR “Men” OR “Male” OR “Males”) AND (“Suicide” OR “Suicides” OR “Suicidal”) AND (“Mental Disorders” OR “Psychiatric Illness” OR “Psychiatric Diagnosis” OR “Behavior Disorders” OR “Mood Disorders” OR “Affective Disorders” OR “Personality Disorders”))	877
Total		13,771

### Study selection

The studies were selected in three phases. In the first phase, as a calibration exercise, the reviewers discussed the eligibility criteria and applied them to a sample of 20% of the studies retrieved, in order to determine the inter-examiner agreement. After achieving a proper level of agreement (Kappa ≥ 0.81), two eligibility reviewers performed a methodical analysis on the titles of the studies, independently. The reviewers were not blind to the names of authors and journals.

In the second phase, the reviewers read the abstracts of the remaining studies, independently. Results in which the titles met the objectives of the study but for which the abstracts were not available were maintained for phase three. Lastly, the studies that had previously been considered eligible, and which were obtained and assessed, were read in full (third phase) to verify whether they met the eligibility criteria.

When the two reviewers disagreed, a third reviewer was consulted to make a final decision. The studies rejected were registered separately, with explanations for the reasons for exclusion.

### Data extraction

After the studies had been selected, they were analyzed by two reviewers, who extracted data independently to gain the following information: authors, location and year of publication, time of assessment, sample characteristics (number and age group), sources of information on attempted or completed suicide, sources of demographic information, mental disorders, outcomes assessed, method for diagnosing mental disorder, prevalence of mental disorders in the group of elderly men who attempted suicide and main result of the study. In order to ensure consistency between the reviewers, a calibration exercise was performed with the two reviewers, in which they extracted the information together from an eligible study. Any disagreement between the reviewers was resolved through discussions and when both reviewers could not agree, a third reviewer was consulted to make a final decision.

The prevalence values for each category of mental disorder, according to the ICD-11, were collected or calculated when required. When calculation of the prevalence and respective confidence interval was required, data regarding the absolute number of individuals with each type of disorder were extracted, along with the total numbers of elderly people with mental disorders and who attempted suicide.

### Individual risk of bias and methodological quality assessment of the studies included

The Joanna Briggs Institute critical appraisal tools for prevalence studies were used to assess the risk of bias among the studies included.^21^ Two authors performed assessments independently, in accordance with the PRISMA-P statement.^18^ Any disagreement between the reviewers was resolved through discussions on the topics assessed, and when the two reviewers could not agree, a third reviewer was consulted to make a final decision.

The questions assessed were the following: Q1) Was the sample frame appropriate for addressing the target population? Q2) Were the study participants sampled appropriately? Q3) Was the sample size adequate? Q4) Were the study subjects and the setting described in detail? Q5) Were the data analyzed with sufficient coverage of the sample identified? Q6) Were valid methods used for identifying the condition? Q7) Was the condition measured in a standard and reliable way for all participants? Q8) Was the statistical analysis adequate? Q9) Was the response rate adequate, and if not, was the low response rate managed appropriately?

Each study was categorized according to the percentage of positive answers in the questions corresponding to the assessment tool. Risk of bias was considered high when up to 49% of the answers were “yes”, moderate when 50% to 69% of the answers were “yes” and low when than 70% of the answers were “yes”.^21^

### Synthesis of results and meta-analysis

A synthesis was performed on the results, with descriptive meta-analysis on the studies included, and this was presented narratively and through tables and figures. The prevalence estimated for each mental disorder considered was calculated using fixed and random-effect models in the meta-analysis, as indicated. The choice of proper effect for correctly representing the results was based on the heterogeneity presented. When heterogeneity was high (I^2^ > 50% or chi-square P-value < 0.05), the random-effects model was selected.^22^

### Quality of evidence-gathering

Quality of evidence and recommendation strength were assessed using the Grading of Recommendation, Assessment, Development and Evaluation (GRADE) tool.^23^ The GRADE pro-GDT software (http://gdt.guidelinedevelopment.org) was used for summarizing the results. This assessment was based on study design, methodological limitations, inconsistencies, indirect evidence, imprecision and other considerations. The quality of evidence was characterized as high, moderate, low or very low.^23^

## RESULTS

### Study selection

**Figure 1.**
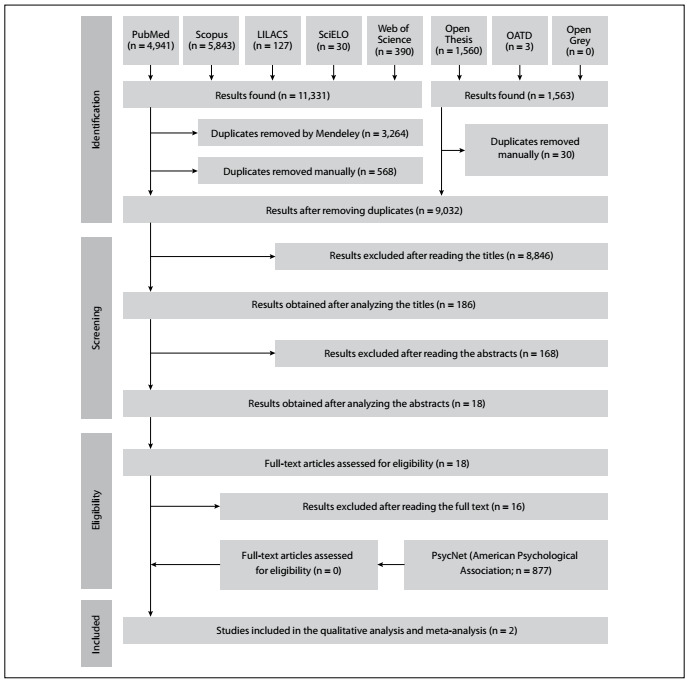
PRISMA flow diagram.

During the first phase of study selection, 12,894 results were found, distributed in eight electronic databases, including the grey literature. After removing duplicate results, 9,032 studies were retained for analysis of titles and abstracts. After this, 18 eligible results were retained for full-text analysis. After reading the full text, 16 of these studies were eliminated because they did not deal only with suicidal ideation or completed suicide and did not present segmented data for men and/or elderly people. Lastly, a database specific to mental health was researched (PsycNet, from the American Psychological Association). Through following the same analysis stages as used in relation to other databases, 877 results were initially identified, but none of them met the inclusion criteria. Thus, in the end, only two studies were selected for qualitative analysis and meta-analysis. The flow diagram shown in Figure 1 describes the process of searching for, identification, inclusion and exclusion of articles.

### Characteristics of eligible studies

The studies were published between 2004 and 2016 and were conducted in Finland^24^ and Australia.^25^ Regarding study design, Almeida et al.^25^ undertook a cohort study while Suominen et al.^24^ did a cross-sectional study. Both studies^24^^,^^25^ reported that ethical approval had been obtained for accessing the diagnostic data and patient characterization data. Almeida et al.^25^ collected data from the Western Australian Data Linkage System (WADLS), based on community-dwelling information, while Suominen et al.^24^ used data obtained from four general hospitals based on records from patients who had been treated for suicide attempts. Although neither of these could be considered to be a balanced epidemiological study, both seem to somehow represent an extended population range. One of the articles^24^ was part of a multicenter study (WHO/EURO Multicentre Study on Parasuicide). However, the definition of elderly participants differed between these two studies: one^24^ defined the age group as over 60 years while the other^25^ defined it as 65 years. For statistical analyses, while Suominen et al.^24^ divided the population studied into two groups (< 60 years and ≥ 60 years), Almeida et al.^25^ considered four groups (65-69 years, 70-74 years, 75-79 years and ≥ 80 years). Additional details are presented in Table 2.

**Table 2.** Summary of the main characteristics of the eligible studies

Author	Study location	Assessment time	Total sample	Age group	Sources of information	Diagnostic method for mental disorder	Mental disorders assessed	Outcomes assessed
Suominen et al.^24^	Helsinki, Finland	1 year (1997-1998)	81	≥ 60	Medical records from four general hospitals in Helsinki	ICD-10	Schizophrenic disorders Mood disorders Substance use-related disorders Neurotic disorders Personality disorders Other mental disorders	Suicide rate within 12 months after hospital release
Almeida et al.^25^	Perth, Australia	16 years (1996-2011)	38,170	> 65	Western Australian Data Linkage System (WADLS)	ICD-8 ICD-9 ICD-10	Depression Bipolar disorder Psychoses Dementia Alcohol-related disorders Substance use-related disorders	Rates of suicide and suicide attempts

ICD = International Classification of Diseases.

### Risk of individual bias of the studies

One of the studies^24^ presented moderate risk of bias (66.6%), while the other^25^ presented low risk of bias (77.7%). The study by Suominen et al.^24^ did not present adequate sample size (Q3), data analysis (Q5) or response rate (Q9). The study by Almeida et al.^25^ did not present adequate data analysis (Q5) or response rate (Q9).

### Synthesis of results and meta-analysis

These two studies presented different results after their statistical analyses had been performed, but it has to be borne in mind that they did not have the same objective. Suominen et al.^24^ observed that there was no statistically significant difference in the number of suicide attempts between the sexes. They also found that only one-fifth of the elderly individuals who attempted suicide were older than 75 years and that most of the elderly subjects had contacted primary healthcare before the attempt. Only 38% of the elderly subjects who attempted suicide for the first time had been diagnosed with mood disorders before the suicide attempt. Among those who made a second suicide attempt, 61% had previously undergone psychiatric treatment.

Almeida et al.^25^ observed that the main variable associated with suicide attempts among the elderly was the existence of previous attempts (hazard ratio, HR 203.14; 95% confidence interval, 95% CI 164.10-251.46). When the data of participants with a history of attempted suicide were removed from the multivariate analysis, bipolar disorder took over as the main associated variable (HR 15.46; 95% CI 9.71-24.62). The elderly men who committed suicide were 3.6 years younger than the elderly men who did not did not die when they attempted suicide.

In the two studies included, a total of 359 elderly people with mental disorders who had attempted suicide were identified. Only three types of mental disorders were reported in both studies and consequently could be synthetized into a single measurement of overall prevalence. Thus, the prevalences of mood disorders (ICD-11: 6A8 and 6CAG.7), schizophrenic disorders (ICD-11: 6A2) and substance use-related disorders (ICD-11: 6C4Z) were ascertained.

The mental disorders observed by Suominen et al.^24^ were personality disorders (3.64%), mood disorders (43.64%), schizophrenic disorders (10.91%), substance use-related disorders (23.64%), neurotic disorders (9.09%) and other mental disorders (9.09%). The results from their study suggested that mood disorders remained undiagnosed within primary healthcare before the suicide attempt.

The mental disorders observed by Almeida et al.^25^ were bipolar disorders (33.57%; 95% CI 23.59-47.79), depression (21.85%; 95% CI 16.61-28.74), schizophrenic disorders (4.71%; 95% CI 1.73-12.84), alcohol-related disorders (12.21%; 95% CI 9.28-16.07) and disorders relating to other substances (3.49%; 95% CI 2.69-4.52). They concluded that previous suicide attempts were associated with new suicide attempt episodes, but not with completion of the act, while psychological disorders were associated with deaths by suicide.

**Figure 2.**
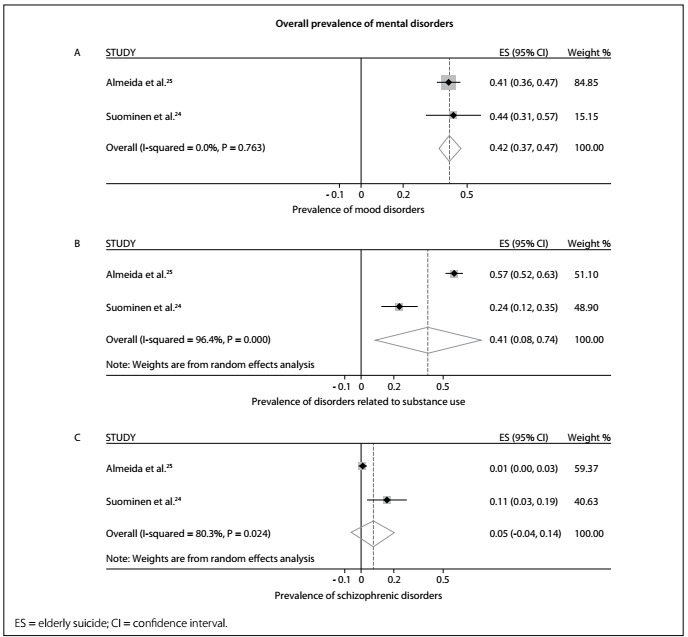
Overall prevalence of each type of mental disorder according to the meta-analyses using fixed and random effects: (a) mood disorders; (b) substance use-related disorders; and (c) schizophrenic disorders.

The pooled prevalence of the mental disorders assessed ranged from 5.0% to 42.0% and the highest prevalence occurred in cases of mood disorders (42.0%; 95% CI 31.0-74.0%; I^2^ 0.0%; P = 0.763), followed by substance use-related disorders (41.0%; 95% CI 8.0-74.0%; I^2^ 96.4; P < 0.001). These estimates were obtained using a fixed-effects model and a random-effects model, respectively. Figure 2a (mood disorders) and Figure 2b (substance use-related disorders) show the measurements for these two disorders. Schizophrenic disorders were the least prevalent (5.0%; 95% CI 0.0-14.0%; I^2^ 80.3%; P = 0.024), as shown in Figure 2c.

In addition to the outcomes assessed in the meta-analyses, other mental disorders were identified, but it was not possible to estimate their overall prevalence, given that data on these other conditions were only presented in a single study.^25^

### Certainty of the evidence identified

The certainty of the evidence identified was divided into levels that were assessed using the GRADE tool.^23^ The certainty level for the outcome relating to the prevalence of mood disorders was classified as low, which means that the true effect may have been substantially different from the estimated effect. Moreover, the certainty level for outcomes relating to substance use-related disorders and schizophrenic disorders was classified as very low, which means that the true effect was probably substantially different from the estimated effect (Table 3). The design of the study was responsible for downgrading by two levels in relation to all outcomes. Moreover, the high inconsistency gave rise to downgrading by two levels in relation to two outcomes (substance use-related disorders and schizophrenic disorders).

**Table 3.** Summary of findings according to the Grading of Recommendations Assessment, Development and Evaluation (GRADE) for the outcomes of the systematic review and meta-analysis

Quality assessment						Summary of results			Significance
Number of studies	Study design	Risk of bias	Inconsistency	Indirectness	Imprecision	Publication bias	Effects	General quality	
Outcome I: Prevalence of mood disorders									
2	Observational	Not severe	Not severe	Not severe	Not severe	None	Fixed (95% CI) 42% (31.0-74.0%)	⊕⊕ Low	Critical
Outcome II: Prevalence of substance use-related disorders									
2	Observational	Not severe	Very severe^1^	Not severe	Not severe	None	Random (95% CI) 41% (8.0-74.0%)	⊕ Very low	Critical
Outcome III: Prevalence of schizophrenic disorders									
2	Observational	Not severe	Very severe^1^	Not severe	Not severe	None	Random (95% CI) 5% (0.0-14.0%)	⊕ Very low	Critical

GRADE Working Group grades of evidence

High certainty: Very confident that the true effect is close to the estimated effect.

Moderate certainty: Moderately confident in the effect estimated. The true effect is likely close to the estimated effect, but it may be substantially different.

Low certainty: Limited confidence in the effect estimated. The true effect may be substantially different from the estimated effect.

Very low certainty: Very little confidence in the effect estimated. The true effect is likely substantially different from the estimated effect.

^1^Downgraded by two levels because of high heterogeneity (I² > 50%).

CI = confidence interval.

## DISCUSSION

Although this systematic review suggested that there was higher prevalence of some types of mood disorders among the elderly people who attempted suicide, the level of certainty for support this statement was limited. It had previously been reported that elderly men presented higher vulnerability towards committing suicide.^24^^,^^26^^,^^27^^,^^28^^,^^29^ This behavior is usually explained in terms of diagnoses of chronic diseases that interfere with quality of life,^30^^,^^31^ or in terms of loneliness, loss of a family member or even boredom or lack of employment.^32^

It is important to consider whether these factors may trigger depression, which would strengthen the results from our systematic review, considering that depression presented significant prevalence among the mental diseases considered in the eligible articles. In this regard, an increasing curve of diagnoses of depression has been observed among elderly people over the last decade, caused mainly by loneliness or feelings of uselessness to society.^33^^,^^34^ Studies conducted in different countries and with different age groups have strongly correlated depression with suicidal tendencies^28^^,^^35^ and have shown that there is a relationship between depression and suicide among elderly men.^25^^,^^36^ Corroborating this information, it has been observed that 70% of elderly people older than 70 years who committed suicide also presented depression.^37^

Another mood disorder that has previously been studied is bipolar disorder. Almeida et al.^25^ showed that elderly people with bipolar disorder were 33 times more likely to commit suicide after the first attempt. These authors^25^ also suggested that, among mental disorders, bipolar disorder increases the likelihood of a second suicide attempt. A correlation between bipolar disorder and suicide has been observed in all age groups, along with a tendency among people with type II bipolar disorder to use more violent and lethal methods in their suicide attempts, compared with individuals with type I.^38^ Suicide among people with bipolar disorder has been found to be influenced by seasonal factors.^39^ Drug treatment is essential to prevent suicide in this population, and use of a combination of mood stabilizers and antidepressants has been correlated with lower risk of suicide.^40^

Schizophrenic disorders were also correlated with higher numbers of suicide attempts in the studies that were considered eligible for the present analysis. Schizophrenic disorders presented the lowest prevalence among the elderly men with mental disorders in this systematic review. These data are concordant with findings in other studies of reduction of diagnostic volume of this type of mental disorder that was observed as age progressed among elderly men.^37^ Elderly men with schizophrenia were found to be 4.71 times more likely to commit suicide^25^ and the number of attempts in this population was strongly correlated with the number of suicides actually committed.^41^ Use of alcohol has also been found to be a strong predictor among schizophrenics indicating that they may commit suicide.^41^ Hor and Taylor^41^ affirmed that the best suicide prevention strategy for schizophrenic patients should be to stimulate them to adhere better to drug therapy. These findings emphasize the importance of timely diagnosis and adequate treatment of mental disorders among elderly men.

It is known that suicide is more prevalent among men.^9^^,^^16^^,^^17^^,^^19^^,^^42^^,^^43^^,^^44^^,^^45^ It affects elderly people at higher rates,^5^^,^^7^^,^^8^^,^^10^^,^^11^^,^^13^ and elderly men use more violent methods of suicide, with emphasis on hanging.^37^ Suicide attempts are therefore a major marker for identifying suicidal behavior, and need to be considered in treatment planning and suicide prevention.^46^ Hence, through presenting the prevalence of suicide attempts for each disorder analyzed, the information collected in our systematic review may be helpful in planning improved care for elderly men and it emphasizes the importance of timely adequate diagnosis for these mental disorders.^38^^,^^39^^,^^47^

Mainly regarding mood disorders, which were more predominantly correlated with suicide attempts in the present meta-analysis, the importance of primary healthcare in determining an early diagnosis needs to be strengthened. If such disorders fail to be satisfactorily diagnosed before the suicide attempt,^24^ this may constitute a significant factor relating to the attempt. Among mental disorders, implementation of the correct drug therapy seems to stand out as the best way to prevent suicide.^40^^,^^41^

Moreover, the number of suicide attempts might be even higher than the estimated number. This underestimation may arise through potential failures in reporting or undervaluation of suicide outcomes because of other diagnoses. Hence, it is important to consider the role of healthcare services in making early diagnoses of mental disorders among elderly men, in order to propose timely and adequate treatment. In cases of attempted suicide, higher levels of care for these patients should be provided, considering that a new attempt may occur or suicide may even be completed.

### Quality of evidence

Using the GRADE tool, the overall quality of evidence was identified as low or very low, depending on the outcomes assessed. This was corroborated by the observational designs of the studies analyzed here, given that such designs generally only attain lower scores when the GRADE assessment tool is used. Moreover, the inconsistency in the prevalence of mental disorders among the studies included downgraded the level of evidence. Lastly, in addition to the potential for underreporting that is usually associated with secondary data on suicide, the quality of evidence is also adversely affected by contextual issues (esteem and pressure in the legal, religious and political environments); diagnostic difficulties in some cases (self-starvation, falls, drowning, car accidents, opiate overdose and euthanasia); and the lack of an internationally standardized procedure for reporting suicide.

### Limitations

Suicide is an important public health problem that affects both developed and developing countries.^1^ In this review, the studies on suicide attempts among elderly men with mental disorders that were included were only conducted in developed countries. Thus, the main limitation of the present study is the low level of certainty of its evidence. Another limitation is the lack of research from countries with emerging economies, which prevents generalization of the data obtained to a global reality. Moreover, the high methodological heterogeneity and the low number of eligible studies suggest that there is a need to conduct further studies with improved designs, to obtain stronger scientific evidence that would lead to more conclusive findings regarding this important topic.

## CONCLUSION

It seems that mood disorders and substance use-related disorders are quite prevalent among elderly men with mental disorders who attempt suicide. Significant imprecision (large prevalence ranges) was associated with low certainty of evidence. Hence, the mean prevalence summaries provided here should be carefully considered, given that real population values may differ substantially from the stated synthetized prevalence. Nevertheless, it seems important to consider the role of healthcare services in making early diagnoses of mental disorders among elderly men, with the aim of diminishing the chances of suicide attempts among them. Since suicide is a multifactorial affliction, the focus should not only be on mental disorders but also be on all factors associated with suicide.
